# Multicellular behaviour enables cooperation in microbial cell aggregates

**DOI:** 10.1098/rstb.2019.0077

**Published:** 2019-10-07

**Authors:** Ali Ebrahimi, Julia Schwartzman, Otto X. Cordero

**Affiliations:** Department of Civil and Environmental Engineering, Massachusetts Institute of Technology, Cambridge, MA 02139, USA

**Keywords:** self-organization, microbial aggregate, alginate, trait-based model

## Abstract

Oligosaccharides produced from the extracellular hydrolysis of biological materials can act as common goods that promote cooperative growth in microbial populations, whereby cell–cell aggregation increases both the per capita availability of resources and the per-cell growth rate. However, aggregation can also have detrimental consequences for growth, as gradients form within aggregates limiting the resource accessibility. We built a computational model, which predicts cooperation is restricted in dense cell aggregates larger than 10 µm because of the emergence of polymer and oligomer counter gradients. We compared these predictions to experiments performed with two well-studied alginate-degrading strains of *Vibrio splendidus*, which varied in their ability to secrete alginate lyase. We observed that both strains can form large aggregates (less than 50 µm), overcoming diffusion limitation by rearranging their internal structure. The stronger enzyme producer grew non-cooperatively and formed aggregates with internal channels that allowed exchange between the bulk environment and the aggregate, whereas the weak enzyme producer showed strongly cooperative growth and formed dense aggregates in which cells near the core mixed by active swimming. Our simulations suggest that the mixing and channelling reduce diffusion limitation and allow cells to uniformly grow in aggregates. Together, these data demonstrate that bacterial behaviour can help overcome competition imposed by resource gradients within cell aggregates.

This article is part of a discussion meeting issue ‘Single cell ecology’.

## 1. Introduction

Microbes live in communities, that is, collectives with thousands of cells influencing each others function and behaviour. In many cases, the activity of the collective can give rise to emergent forms of behaviour and spatial organization that increase the fitness of its members. For instance, the predatory bacterium *Myxococcus xanthus* and the social amoeba *Dictiostelium discoideum* form multicellular groups to forage for food and to differentiate into stalked fruiting bodies that propagate spores through the air [1]. Likewise, the multispecies bacterial biofilm communities of the oral cavity, ammonia oxidizing or nitrogen-fixing bacterial consortia, and methane oxidizing archaea exhibit complex structure that may create proximity between taxa optimal for the growth of individuals [2–6]. In all these examples, the structure of the collective provides information about its function. How such structures emerge and what processes regulate their dynamics remain open questions.

Polymer-degrading microbes are the primary recyclers of dead organic matter in the biosphere [7], and often form dense communities. Organisms in these collectives confront the problem of digesting objects that are insoluble and, in many cases, much larger than their membrane transporters. To deal with this problem, polymer-degrading bacteria have evolved strategies that turn the extracellular environment into a digester: large substrates are hydrolysed by secreted enzymes and hydrolysis products captured before they are lost to diffusion, fluid flow or competitors. Examples of this phenomenon are found in the degradation of proteins, mediated by secreted proteases [8] or complex carbohydrates, such as alginate, mediated by secreted alginate lyases [9,10]. In general, primary producers such as phytoplankton, plants, photo- and chemo-synthetic microbes store most carbon and energy in complex polymeric materials. Therefore, the secreted hydrolytic enzymes of polymer-degrading microbes play a central role in the recycling of organic matter from primary production and the closing of element cycles in ecosystems.

One of the most interesting consequences of extracellular enzyme activity is the potential for the so-called cooperative growth dynamics to emerge. Cooperative growth implies that the per capita growth rate is not constant, as in classical exponential growth, but positively dependent on cell density [8]. A simple explanation for these dynamics is that when populations obtain resources via extracellular enzymes they can facilitate their own growth: cells secrete enzymes, which release oligomers, which are used to produce cells, thereby creating a positive feedback loop. This effect is most pronounced when cells are able to recover only a small fraction of the oligomer released by their enzymes, so that having another cell in the neighbourhood increases the local concentration of oligomers and the uptake rate [11]. Moreover, dense aggregates such as biofilms can increase the retention of hydrolysis products, further increasing local concentrations [12]. When these conditions are met, cells that form aggregates should grow at faster rates than those that remain isolated. This is because within cell aggregates the concentration of local hydrolysis products is higher, thus increasing the growth rate of oligomer-limited cells. Therefore, cooperative growth implies that, as a collective, populations can recover more of the product thereby increasing per capita growth rates.

Although aggregation allows cooperation between cells, close cell–cell packing can also become detrimental once competition for oligomers overrides the benefits of cooperation. Moreover, in a structured environment, growth processes can also lead to the formation of resource gradients [13–15], which create areas of low resource supply that emerge because of diffusion limitation and rapid consumption of nutrients. This suggests that at certain aggregate densities, the ‘returns’ from aggregation diminish, thus limiting the size that aggregates can reach. Interestingly, some microbial species have been shown to have the ability to differentiate their phenotypes within cell aggregates. For example, *Pseudomonas aeruginosa* populations spatially segregate into fast-growing planktonic, and slow-growing biofilm sub-populations when grown in rich medium [16]. However, the potential for dynamic rearrangements within cell polymer-degrading aggregates, and the consequences of rearrangement for aggregate structure and function have not yet been explored.

We seek here to understand the conditions that lead to beneficial aggregation among polymer-degrading cells and the potential for cell reorganization to enhance aggregate growth. To address these questions, we developed an agent-based physical model of self-organized aggregates formed during growth of microbial populations on a soluble polymer. We used this model to explore how the aggregate geometry constrains the cellular uptake of nutrients and growth. We tested some of the predictions of our model using alginate-degrading isolates of the marine microbe *Vibrio splendidus* [9,17–20]. The two alginate-degrading strains secrete different amounts of hydrolytic enzyme and have different requirements for cooperation, prompting us to ask how bacterial behaviour in the population alters the structure of aggregates and the efficiency with which alginate is consumed by cells. We find that the benefit of aggregation is negatively related to the hydrolytic power of individual cells, and that novel aggregate forms, such as ‘channelling’ and ‘mixing’ emerge by rearrangement of cells, as design solutions that maximize carbon use efficiency.

## 2. Results

**Figure 1.**
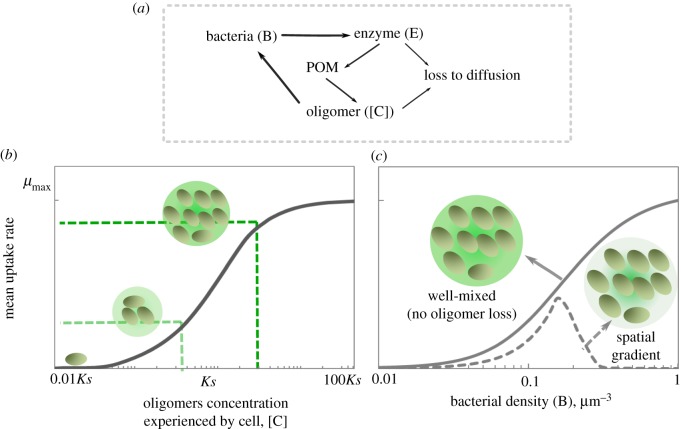
Bacterial aggregation in polymeric substrates leads to cooperative growth and enhances per capita uptake rate. (*a*) Conceptual representation of our mathematical model: bacterial cells secrete enzymes that break down polymers, releasing oligomers which are taken up by bacterial cells, thus closing the loop. Enzymes and oligomers can be lost to diffusion. (*b*) The mean cellular uptake rate is a function of oligomer concentration, represented by a Monod model of growth kinetics. A schematic of the effects of initial cell numbers on oligomer concentration and its corresponding uptake rates are shown. (*c*) Mean uptake rate by bacterial cells are mathematically simulated as a function of initial cell density within a spherical aggregate of diameter 20 µm, 20 h after inoculation. The simulation results are shown for a well-mixed closed system with no oligomer loss (solid line) and for an unmixed aggregate where diffusion is the dominant transport mechanism that allows for the loss of oligomers to the bulk environment at the aggregate periphery (dashed line). At low bacterial densities, the aggregate behaves as a well-mixed reactor of size 20 µm, indicating the absence of gradients. (Online version in colour.)

In our model, as in our experiments, a cell aggregate is immersed in a polymer solution. The polymer (soluble alginate) can penetrate the aggregate by diffusion, where it interacts with enzymes (alginate lyases) that hydrolyse it, releasing oligomers that can be transported into cells (figure 1*a*). Oligomers can diffuse and be consumed by cells based on Monod kinetics (figure 1*b*). The interplay between these processes gives rise to spatial gradients that can influence cell growth and depend on aggregate density and size. To study the impact of aggregate density on the growth rate of individuals within an aggregate, we fixed the aggregate radius to 20 µm and measured the mean rate by which individual cells take up oligomers, as a function of increasing bacterial density (figure 1*c*). In our model, soluble polymers are assumed to be at a concentration of 0.1 mg l^−1^. Alginate lyase activity is assumed to be diffusion limited, as can happen if the enzyme is tethered to the cell membrane [9,21] or trapped in the extracellular matrix of a biofilm [12].

### (a) The balance between cooperation and competition

The computational results revealed an optimal cell density within the aggregate that maximized mean per capita growth (figure 1*c*). At densities below the optimum, the per capita growth rate increased with cell density. The positive density dependence observed below the optimal cell density is consistent with the benefit derived from taking up oligomers released by neighbours, which would otherwise get lost to diffusion. Thus, below the optimal cell density, cells cooperate by sharing the oligomers released by their enzymes. For this positive dependence between uptake (growth) and cell density to emerge, an increase in the oligomer concentration should increase the per capita uptake rate. Because in our simulations, the relationship between oligomer uptake rate and oligomer concentration followed Monod kinetics (figure 1*b*), the conditions for positive density dependence were those where oligomer concentration was approximately equal to the half-saturation of the Monod curve, *Ks*, that is, the affinity of cells to oligomers. By contrast, when the concentration of oligomers was significantly higher than *Ks* the uptake rate saturated, limiting the benefit of increasing cell density on carbon uptake. From this point on, an increase in cell density was detrimental for the per capita uptake (figure 1*c*). In these conditions, competition for substrate overrides the benefits of cooperation. Note that *Ks* is a phenotypic property of the cells that is measured at the level of the whole population and equals the concentration of growth limiting substrate that supports half of the maximum growth rate.

**Figure 2.**
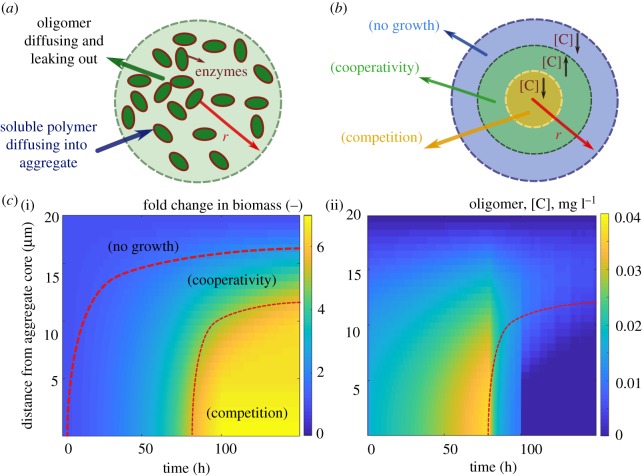
Spatial gradients restrict oligomer uptake by bacterial cells in aggregates. (*a*) Schematic of the individual-based model developed for bacterial growth in an aggregate. The boundary condition for diffusion of oligomers is set to be zero at the aggregate periphery and soluble polymer is considered to be present at the aggregate surface at a constant concentration. Enzymes are assumed to be membrane bound with no diffusion. (*b*) Schematic of the narrow range of optimal oligomer concentration, predicted by the model, that allows for cooperative bacterial growth in aggregates. (*c*) The fold of change in biomass (i) and oligomer concentration (ii) are shown along the aggregate radius over time, revealing the migration of a zone that optimizes biomass production and the emergence of competition within the core of the aggregate. The simulations are performed for an aggregate with constant radius of 20 µm and initial cell density of 0.2 µm^−3^. The enzyme production rate is assumed to be 0.02 h^−1^.

To better understand the impact of cell density on cell growth, we simulated the emergence of spatial gradients within aggregates of fixed size (20 µm) and low initial cell density (0.2 µm^−3^), using the individual-based model (figure 2*a*). Our simulation results revealed that counter gradients of polymer (diffusing from the outside; electronic supplementary material, figure S1) and oligomer (diffusing from the inside), led to a narrow range of positions along the radial axis of the aggregate for which cooperative growth took place (figure 2*b*). Outside this narrow band, towards the core of the aggregate, cells quickly experienced strong competition owing to the high cell densities attained and the slow diffusion of oligomers towards the core (figure 2*c*). Towards the periphery of the aggregate, most oligomers released by enzymatic activity were lost by diffusion to the bulk environment, limiting the growth of cells. This lead to a situation where cooperative cells were ‘sandwiched’ in between cells starved by the losses imposed by diffusion and by competition (figure 2*b,c*). Our model shows that, contrary to intuition, the more public good the cells make available, the faster the transition to competition occurs when cells are in a densely packed aggregate. This is because increasing the per capita activity of secreted enzymes lowers the cell density threshold where cooperative growth transitions towards competition for resources. Effectively, the growth rate saturates when fewer cells are present (electronic supplementary material, figure S2), and because diffusion of polymer from the periphery limits growth, hydrolysed product is only available at the periphery (electronic supplementary material, figure S2). Based on these results, we predict that aggregation supports cooperative growth only for a narrow range of cell densities (between 0.1 and 0.4 cells µm^−3^) and cells with low enzyme production rates (electronic supplementary material, figure S3).

### (b) Multicellular behaviour allows individuals to avoid competition

**Figure 3.**
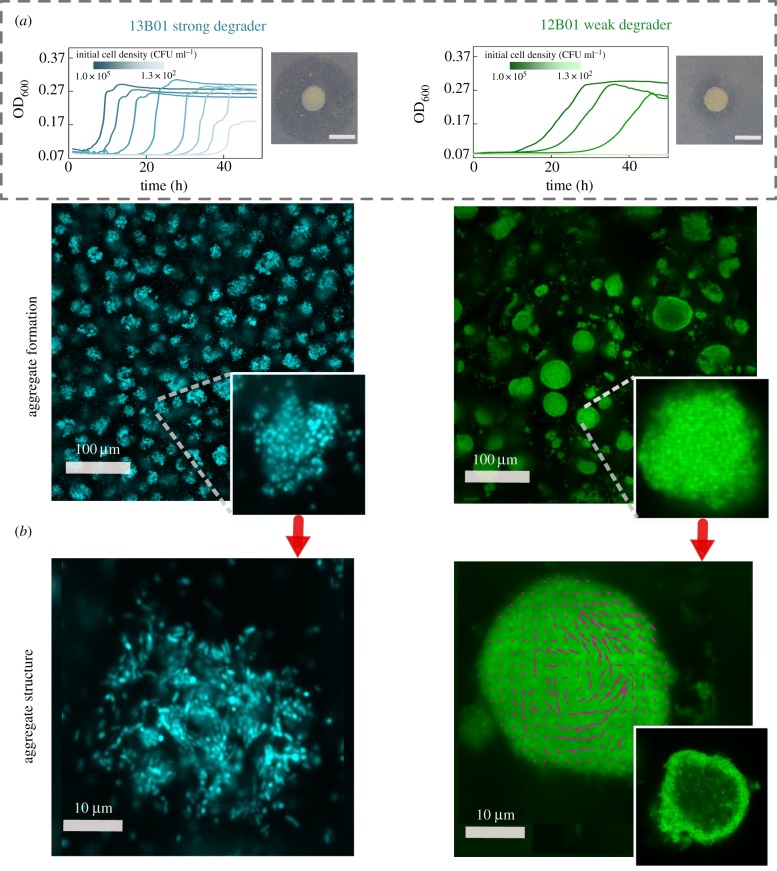
Bacterial enzymatic activity affects bacterial cooperative growth and resulting aggregate structure. (*a*) Density-dependent growth for 12B01 and 13B01. Experiments were performed to measure the growth of these two strains in well-mixed liquid alginate as a function of the initial cell density. In the absence of density dependence, we expect growth curves to be delayed in proportion to the dilution factor, as in the case of 13B01. By contrast, if cells grow cooperatively, we expect delays that grow disproportionally with dilutions—or no growth whatsoever, as observed in 12B01. The broadcast alginate lyase activity of 12B01 and 13B01 is shown on the right of each panel, measured as halos on cetylpyridinium chloride-treated alginate agar plates. The dark grey halo indicates the degree of broadcast alginate lyase activity originating from colonies of the two strains after 48 h of growth. (*b*) Auto-aggregation of 13B01 and 12B01. The aggregate structure is shown as a maximum-intensity projection of 100 µm image stacks, and a cross section taken in the middle of the aggregate is shown in the inset. Confocal images were taken after 24 h of incubation with 0.07% (w/w) alginate with shaking at 25°C. A section taken at mid-plane (right) from 28 h old aggregates reveals that dense 12B01 aggregates undergo restructuring. Magenta vectors superimposed on 12B01 indicate the average velocity of cells over five frames (500 ms). The images were visualized at 40× magnification and 10× optical zoom was applied to highlight single aggregates. (Online version in colour.)

**Figure 4.**
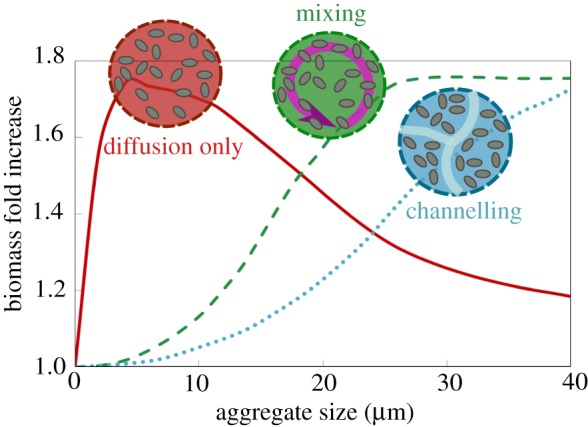
Bacterial reorganization within aggregates optimizes biomass accumulations in bacterial aggregates. Simulation results for biomass accumulation as a function of aggregate size are shown for the two dominant aggregation structures that we experimentally observed (figure 3): mixing (dashed line), channelling (dotted line). The reference scenario with no biological strategy, diffusion governed, is represented for comparison (red line). Aggregate size is shown at 24 h after inoculation. An initial cell density of 0.2 µm^−3^ and the enzyme production rate of 0.02 h^−1^ are assumed. (Online version in colour.)

Our model is based on a number of simplifying assumptions, such as cells are not able to move within the aggregate and remain uniformly packed. In reality, however, cells may have behavioural or physiological strategies to cope with the limits of diffusion within aggregates. To study possible aggregation strategies, we performed experiments in which we induced auto-aggregation of marine bacteria capable of degrading alginate. In particular, we focused on two strains of alginate-degrading *V. splendidus*, 12B01 and 13B01. The two strains were chosen because 13B01 has about 10 times higher alginate lyase broadcasting ability (represented as halo area, figure 3*a*) [17] during growth on alginate polymer compared to 12B01, leading to different hydrolysis kinetics [9]. Moreover, we found that the weak enzyme broadcaster (12B01) exhibited positive density-dependent growth when cultured with 0.1 mg l^−1^ alginate as a sole carbon source. That is, we observed no growth when cells were inoculated below a threshold cell density, indicating that growth was positively dependent on population density (cooperative growth). By contrast, the strong enzyme broadcaster (13B01) did not display signs of density dependence, suggesting a much weaker tendency to cooperate. Both cultures contained cell aggregates, which appeared in absorbance readings as fluctuating measurements beginning in late exponential growth phase. Such fluctuations arise from the variability in the absorption and scattering of light by objects of different size. To better understand these aggregates, we set up a culture system where we could periodically sub-sample cells as aggregate formation developed (electronic supplementary material, figure S4). To induce auto-aggregation, we incubated an initial population of 10^4^ CFU ml^−1^ in 70 ml 0.075% low-viscosity alginate in 250 ml shaking flasks (electronic supplementary material, figure S4). Consistent with our model predictions, we observed that the weak enzyme producer (12B01) formed aggregates with significantly higher bacterial density (approx. 0.75 cells µm^−2^) compared to the strong enzyme producer (13B01 approx. 0.3 cells µm^−2^) (figure 3*b,c*). 12B01 formed densely packed aggregates (figure 3*c*), consistent with the notion that low hydrolysis rate per cell requires more cooperation. On the other hand, the loosely packed aggregates of 13B01 had a structure that resembled balls of crumpled paper, with folds and facets containing seemingly aligned cells (figure 3*b*). Time lapses of 13B01 aggregates revealed that cells continuously rearranged themselves on the aggregates through frequent attachment and detachment (electronic supplementary material, Movie S1). We speculated that these structures, which ranged between 2 and 10 µm in diameter, might represent a network of flow channels within the aggregates that promote the migration of detached cells, polymer, and hydrolysis product between clusters (electronic supplementary material, Movie S2 and figure S5). Simulations showed that channels with an average size of 6 µm in diameter improved uptake rates in large aggregates (greater than 20 µm), while providing no benefit in small aggregates (less than 10 µm; figure 4). Therefore, the channelled structures formed by 13B01 may overcome diffusion limitation and facilitate exchange between the aggregate interior and the bulk environment.

Despite the dense packing of the weak degrader 12B01, aggregates of this strain showed an average size of approximately 20 µm in diameter, with a maximum of more than 50 µm (electronic supplementary material, figure S6). This prompted us to hypothesize that the behaviour of individual cells within the 12B01 aggregate might promote rearrangement along the radial axis, a strategy that would overcome the limits of diffusion and delay the onset of competition at the aggregate core. To measure behaviour within the aggregate, we recorded time-lapse images over periods of 10 min at a fixed cross section of the aggregate core. Surprisingly, we found that there was rapid mixing within the core of the dense aggregates formed by the weak degrader 12B01. This mixing emerged when the aggregates exceeded 15–20 µm in diameter (figure 3*b*; electronic supplementary material, Movie S3). The hollow structures that emerged are reminiscent of the characteristic hollowing of surface attached biofilms that is observed during cell dispersal [22–24] (electronic supplementary material, Movie S4).

Complementing our observations with mathematical modelling of the flow patterns within the aggregate core demonstrated that mixing in the centre of an aggregate can enhance resource acquisition in large aggregate sizes by homogenizing gradients (figure 4). Collectively, these experimental observations and simulations suggest that hollowing and channel formation may be additional ways for cell aggregates to overcome size constraints imposed by resource gradients. Future work will be needed to better understand how different physiological attributes of individuals give rise to particular aggregate architectures, and how these forms shape the function of the collective in natural environments.

## 3. Discussion

A known advantage of dense population clustering is that it allows cells to cooperate by sharing public goods. During growth on complex substrates such as polymers, high cell densities locally concentrate enzymatic activity in a small area (aggregates), increasing the local concentration of hydrolysed oligomer [25]. In this study, we asked what physical and physiological constraints limit cooperative growth in aggregates, and how variation in the amount of enzymatic activity expressed by individual cells might give rise to structural variation of the collective. Our work demonstrates that the ability of cell aggregation to support cooperative growth is limited by counter gradients of polymer and oligomer, which are formed by diffusion and consumption (figure 1). These counter gradients define a narrow zone within an aggregate where cooperative growth may be supported (figure 2). Thus, hydrolysis and consumption of oligomers paired with substrate diffusion set limits on the size and the density of self-organized aggregates.

A key finding of our simulations and experiments is that the limitations of growth in colonies can be overcome by multicellular behaviours such as the formation of channelled aggregates or by mixing within the aggregate core (figure 3*b* and the electronic supplementary material, Movies S2 and S3). Such behavioural traits prevent the formation of gradients and the rise of competitive dynamics within aggregates. In particular, we observed that physiological variation in enzyme secretion between the two strains is correlated to two distinct strategies that our simulations predict mitigate competitive growth dynamics. In particular, growth dynamics in crumpled paper aggregates, formed by cells that express high levels of alginate lyase, are optimized by channels and by dispersal-driven exchange to and from the aggregate. By contrast, in densely packed aggregates, formed by cells that express low levels of alginate lyase, cooperative growth can be sustained if cellular motility mixes the centre of densely packed aggregates (electronic supplementary material, Movie S1) and forms a hollow interior (figure 3*b*). Directional ‘flows’ of motile cells similar to those we observe in the aggregates have been previously reported as an emergent property of dense, confined suspensions of motile bacterial cells [26]. While ‘hollowing’ has previously been reported in the context of the seeding dispersal of mature surface-associated biofilms [27,28], our work suggest that mixing within an aggregate hollow is a strategy to avoid competition at the aggregate core in the context of polymer-degrading colonies.

Despite the importance of cell–cell aggregation in natural environments [29,30], and a molecular understanding of the mechanisms by which diverse microbial taxa adhere to surfaces [31] and to each other [4], fundamental gaps remain to understand how emergent structures arise from the interactions between individual cells in a collective, and how such forms reflect the collective function of populations. While multiple physio-chemical mechanisms have been suggested that promote cell–cell aggregation [32–35], little effort has been devoted to linking physiological processes such as phenotypic differentiation [36,37], motility [38], quorum signalling [39], to the formation of emergent structures by microbial populations in aggregates. Linking cellular processes to the formation of complex spatial structure is an important step to understanding how populations and communities of microbes assemble and function. Future work focusing on the quantification of individual cell physiology *in situ* will be needed to understand how single-cell physiology determines multicellular structures and how the emerging structures feed back on individual's behaviour.

## 4. Methods

### (a) Mathematical modelling of bacterial aggregates

The mathematical model describes individual cell activity within spherical aggregates in the presence of radial chemical gradients. We developed an agent-based model to quantify single-cell interactions with polymeric substrates including enzyme secretion, uptake of breakdown products, growth and division. The polymeric substrates diffuse into the aggregate from the aggregate periphery. The model assumes well-mixed conditions with no accumulation of chemicals in the bulk environment (mimicking an open system) and thus the concentration of polymer and oligomer at the aggregate periphery is modelled as a constant concentration. The oligomer concentration is assumed to be zero at the periphery, so bacteria depend on polymer degradation within the aggregate to grow. Individual cells are uniformly distributed in the spherical domain and consume substrate and grow in response to local oligomer concentration. In our simulations, cells are not motile. A more detailed description of the model is provided in the electronic supplementary material.

### (b) Bacterial aggregate formation experiments

Experiments were performed in shaken flasks. Each 250 ml flask contained 70 ml of a defined minimal media supplemented with 0.075% (w/v) low-viscosity alginate soluble from brown algae (Sigma-Aldrich, A1112) and bacterial cells at an A_600_ of 1.0 were diluted 10^−3^. Flasks were incubated at 25°C, shaking at 200 r.p.m. A low alginate concentration was used to avoid changing the viscosity of the medium and to reduce passive aggregate formation owing to depletion interactions [30]. To visualize bacterial aggregates and their spatial structures, 200 µl subsamples were stained with the DNA-intercalating dye SYTO9 (Thermo Fisher, S34854) at a 1 : 285 dilution in 96-well plates with optically clear plastic bottoms (VWR 10062-900). To avoid evaporation from the wells, sterile self-adhesive sealing films were used to seal the 96-well plates. Additional experimental methods are described in the electronic supplementary material.

## Supplementary Material

Supplementary Information

## Supplementary Material

Movie S1 - single cell behavior around aggregates

## Supplementary Material

Movie S2 - 13B01 aggregate

## Supplementary Material

Movie S3 - Mixing within 12B01's aggregates

## Supplementary Material

Movie S4 - Hollowing of 12B01's aggregates

## Data accessibility

The MATLAB codes for mathematical modelling of bacterial aggregates are deposited at https://github.com/alieb-mit-edu in repository for Bacterial Aggregate Model (BAM Ver.1).
