# Supplementary material for: Multicellular behaviour enables cooperation in microbial cell aggregates
Source: Philos Trans R Soc Lond B Biol Sci. 2019 Oct 7;374(1786):20190077. doi: 10.1098/rstb.2019.0077 (PMC6792450; doi:10.1098/rstb.2019.0077)
Supplement: Supplementary Information [file rstb20190077supp1.pdf]

Supplemental Information

Supplemental Figures

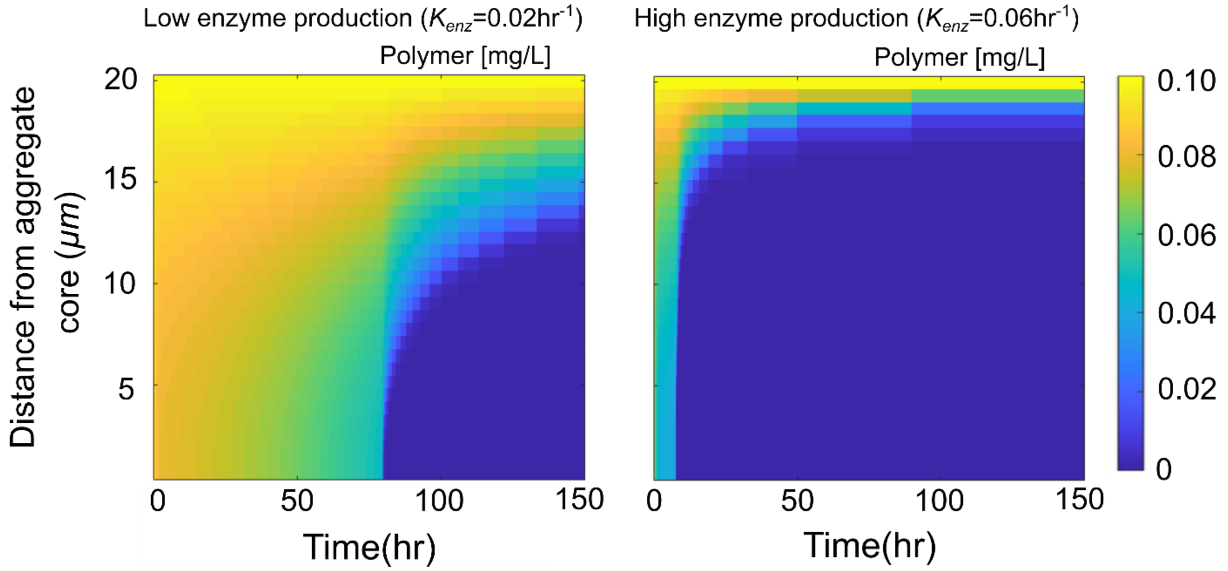

Figure S1. Spatial gradients of polymer concentration along aggregate radius over time for two enzyme production rates ($K_{enz.}$), respectively. The simulations are performed for an aggregate with constant radius of 20 μm and initial cell density of 0.2 μm^{-3}.

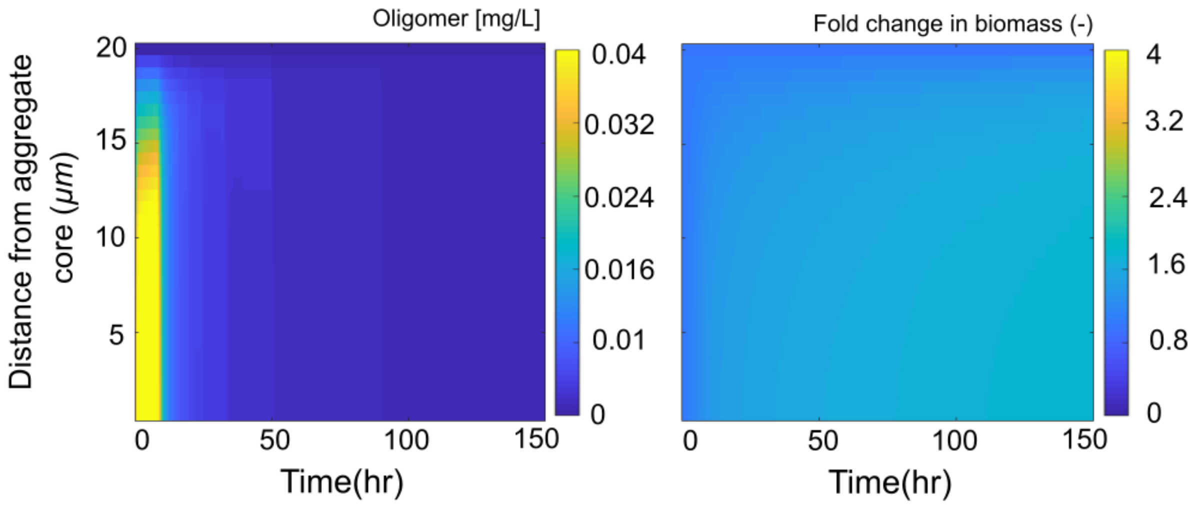

Figure S2. Oligomer concentration and fold change in biomass along the aggregate radius over time are shown for high enzyme production rates ($K_{enz.}$=0.06 hr^{-1}). The simulations are performed for an aggregate with constant radius of 20 μm and initial cell density of 0.2 μm^{-3}.

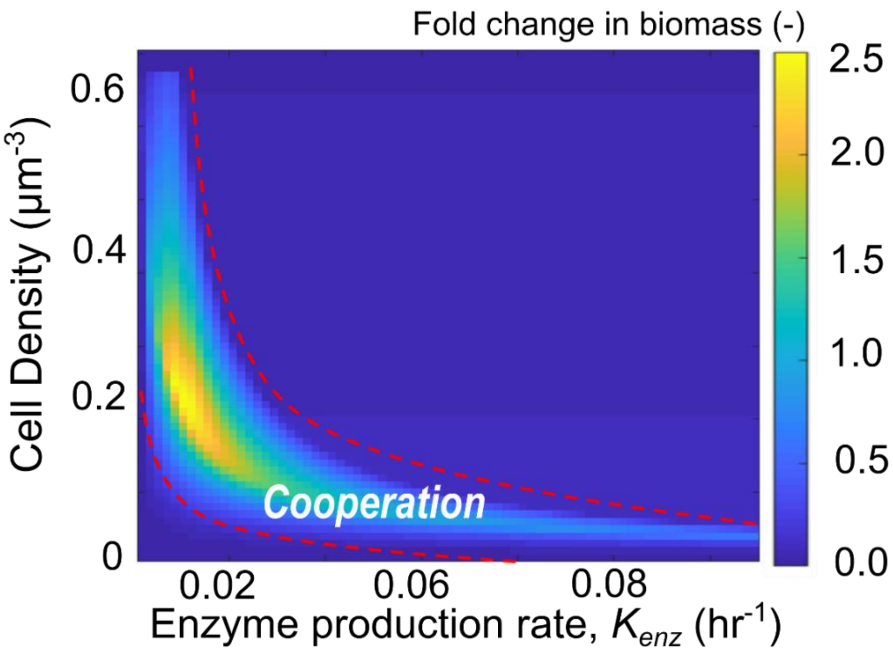

Figure S3. Fold change in biomass as a function of initial cell density and enzyme production rate are shown. Individual simulations are performed by assigning different values of cell density and enzyme production rates for an aggregate with a constant size (20 μm in radius). The results are shown for after 70 h. The fold change in biomass is averaged over the aggregate radius.

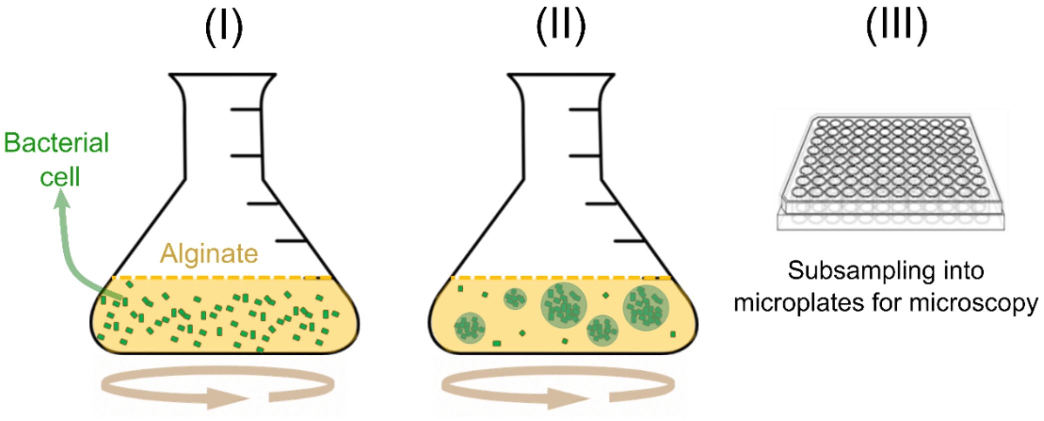

Figure S4. Experimental setup for monitoring aggregate formation and structure. Soluble low viscosity alginate is used as the main carbon source. Bacterial cells at low initial cell density (10^4 CFU/mL) are used in shaking flasks with 70 mL solution. Subsamples of 200 μL from flasks are taken into 96 microplates for microscopy.

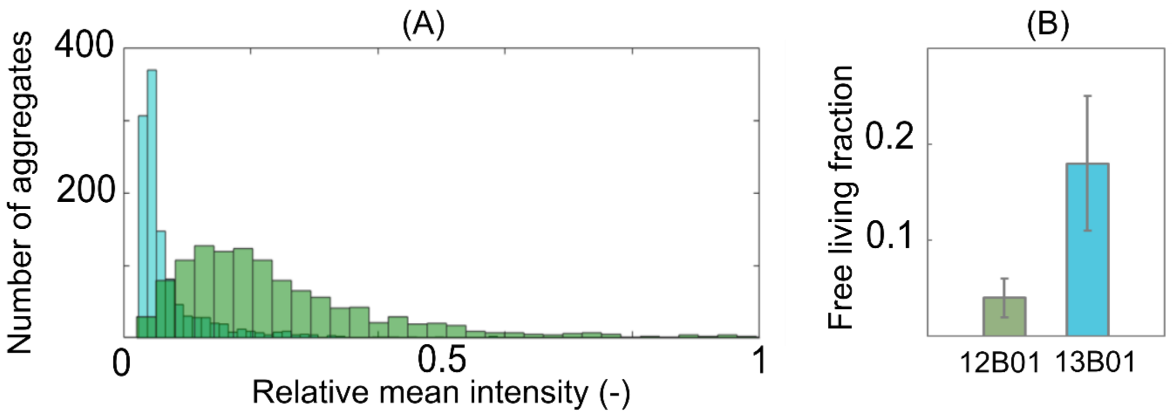

Figure S5. (A) Bacterial cell density distribution within aggregates, represented as relative florescent intensity. Over 1000 aggregates are analyzed. (B) Fraction of free living cells is shown after 24 h from incubation.

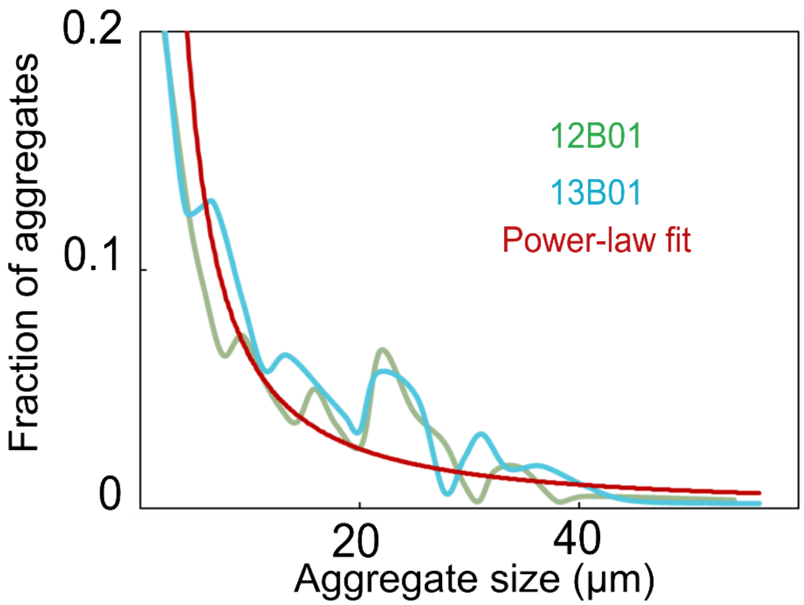

Figure S6. Quantification of aggregate size distributions for two Vibrio strains (12B01 and 13B01). Aggregates after 24 h from inoculation are measured. 250 aggregates are visualized uniform randomly over different fields of views of microscope.

Movie Captions

Movie S1. Dispersal and attachment/detachment of cells around aggregates of 13B01. The frame rate is twice the real time.

Movie S2. Channeling strategy in aggregates from 13B01. The frame rate is twice the real time.

Movie S3. Mixing strategy in aggregates from 12B01. The frame rate is twice the real time.

Movie S4. Hollowing and dispersal strategy in large aggregates of 12B01. The frame rate is twice the real time.

Supplemental Methods

1. Experimental Methods:

1.1 Culture conditions

Bacterial strains used in this study were previously isolated by plating samples from size fractionated water samples [9–11]. For culturing from glycerol stocks, strains were streaked onto Marine Broth 2216 (Difco 279110) 1.5% agar (BD 214010) plates. Single colonies were picked and transferred to 3 mL liquid Marine Broth 2216 in an 18 mm test tube and incubated at 25ºC, shaking at 200 rpm to establish seed cultures. Seed cultures were harvested after ~5 hours by centrifugation for 1 min at 10000 rpm (Eppendorf 5415D, Rotor F45-24-11). The supernatant was discarded and serial dilution of these cells were used to establish pre-cultures in pH 8.2 minimal medium supplemented with 20 mM glucose (see below for full recipe). Following overnight growth, cell density was measured in 1 cm cuvettes by absorbance measurements at 600 nm (A_{600}) using a NanoDrop Spectrophotometer (ThermoFisher Scientific). Cultures with absorbance between 0.5-0.7 were used for experiments measuring positive-density dependent growth and aggregate formation.

1.2 Halo assays to measure alginate lyase activity

Alginate lyase activity was detected as described previously (1), with minor modification. Briefly, assay plates were prepared by adding filter-sterilized 62.5 mL 2.5 % low viscosity alginate to an autoclaved bottle of 500 mL marine broth containing 1.5 % agar (Difco) cooled to 50 °C. Plates were dried overnight, and 5 µL of cultured cells, prepared as described above, were spotted onto the plates. After 48 h of incubation at 50 °C, plates were flooded with a 10 % w/v solution of cetylpyridinium chloride (CP) and deionized water. CP s a cationic quatrinary ammonium compound that adsorbs to polymeric alginate, revealing undigested alginate remaining in the agar as an opaque haze. The plates were incubated with the CP for 20 minutes static at room temperature, at which point the solution was decanted. The plates were gently flooded with deionized water, so that colonies were not disturbed, and the water was exchanged 3 times to remove excess CP. Images of the colonies and 'halos' where polymeric alginate had been digested were obtained using a Cannon EOS Rebel T7.1 digital camera with a EF-S 35 mm f/2.8 IS STM Macro lens.

1.3 Minimal Medium

4x Seawater

Component	Amount (per L)	FW (g/mol)	Concentration (mM)
NaCl	80 g	58.44	1369
$MgCl_2$-$6H_2O$	12 g	203.20	59

$CaCl_2$-$2H_2O$	0.60 g	147.02	4
KCl	2.0 g	74.56	27

Adjust to 1 L with milliQ water and filter sterilize through 0.2 µm.

<u>1000x Trace Minerals (per L)</u>

Dissolve in 20 mM HCl (to avoid precipitate):

**Substance**	**mg / L**
$FeSO_4 * 7H_2O$	2100
H_3BO_3	30
$MnCl_2 * 4H_2O$	100
$CoCl_2 * 6H_2O$	190
$NiSO_4 * 6H_2O$	2.2
$CuSO_4$	2.7
$ZnSO_4 * 7H_2O$	144
$Na_2MoO_4 * 2H_2O$	36
$NaVO_3$	25
$NaWO_4$ $2H_2O$	25
SeO_2	2.5

**Note*: $NaVO_3$ and $NaWO_4 * 2H_2O$ should only be opened in the hood.

Filter sterilize through 0.2 µm.

For long-term storage, freeze at -20 C in aliquots of 1 mL. Protect from light.

<u>1000x Vitamins</u>

Dissolve in 10 mM MOPS, pH 7.2:

**Substance**	**mg/L**
Riboflavin	100
D-Biotin	30
Thiamine hydrochloride	100
L-ascorbic acid	100
Ca-d- pantothenate	100
Folate	100
Nicotinate	100
4-aminobenzoic acid	100
pyridoxine HCl	100
Lipoic acid	100

NAD	100
Thiamin pyrophosphate	100
Cyanocobalamin	10

Titrate with a couple of drops of 5 M NaOH to avoid precipitate.
Filter sterilize through 0.2 µm.
Store at 4 C in the dark. Stable for a couple months.
For long-term storage, freeze at -20 C in 1 mL aliquots. Protect from light.

Nitrogen source

1 M ammonium chloride (100x)
Dissolve 2.14 g NH_4Cl in 40 mL distilled water.
Filter sterilize through 0.2 µm.

Phosphorus source

0.5 M phosphate dibasic (500x)
Dissolve 2.84 g Na_2HPO_4 in 40 mL distilled water.
Filter sterilize through 0.2 µm.

Sulfur source

1 M sodium sulfate (1000x)
Dissolve 5.68 g Na_2SO_4 in 40 mL distilled water.
Filter sterilize through 0.2 µm.

HEPES buffer:

1 M HEPES buffer (20x), pH 8.2

Per 100 mL

26.29 g HEPES sodium salt
Dissolve in 75 mL distilled water. Adjust pH to 8.2 with concentrated NaOH and constant stirring. Bring final volume to 1 L with water. Filter sterilize through 0.2 µm. Store at 4 C.

Basic recipe for carbon-free medium (500 mL)

0.5 mL vitamins
0.5 mL trace metals
0.5 mL 1000x sodium sulfate
1.0 mL of 500x phosphate dibasic
5 mL of 100x ammonium chloride

25 mL of 20x HEPES buffer, pH 8.2
50 mL of 4x seawater
416.5 mL of sterile milliQ H_2O

Glucose stock:
1 M glucose (50x):
Dissolve 1.8 g D-glucose in 10 mL carbon-free minimal medium.
Filter sterilize through 0.2 µm. Store at 4 C.

Preparation of glucose minimal medium:
Add 2 mL glucose stock to 98 mL carbon-free minimal medium.

Preparation of alginate minimal medium:
0.1% Low viscosity alginate (Sigma A1112)
Add 0.1 g alginate to 100 mL sterile carbon-free minimal medium. Add slowly with stirring: heat <100 °C can help the polymer hydrate and dissolve.
Filter sterilize through 5 µm Sterivex filters.

1.4 Measurement of positive-density-dependent growth

To compare the ability of the two strains used in this study to grow on polymeric alginate, cells were grown as described above, then pelleted, and re-suspended at an optical density of 1.0. A multichannel pipette was used to transfer 150 µL of 1 g/L alginate minimal medium to the wells of a 96-well plate. These washed, concentrated cells were diluted 10^{-3} in 1 g/L alginate minimal medium, and 100 µL was added to the first column of a 96-well plate. A multichannel pipette was used to mix the wells in the column, and aliquot 50 µL of medium to the subsequent column. This established 3-fold dilutions of the initial cell population. The 96-well plate was sealed with optically clear sealing tape, and the plate was incubated with double-orbital shaking at 25 C in a Tecan Spark plate reader. Absorbance (600 nm) was measured in 15-minute intervals. These measurements were used to test for the existence of a minimum cell density that supported growth.

1.5 Confocal microscopy and image processing:

Microscopy was performed on micro-confocal high-content imaging system (ImageXpress Micro Confocal, Molecular Devices), using the 60 µm pinhole spinning disk mode. Fluorescent signal was visualized with a LED light source (Lumencore Spectra X light engine), bandpass filters (ex 482/35 nm em 538/40 nm dichroic 506 nm), at 40x magnification (Nikon Ph 2 S Plan Fluor ELWD ADM 0.60 NA cc 0-2 mm, correction collar set to 1.1), and a sCMOS detector (Andor Zyla). To visualize

aggregates,100 µm image stacks sampled at 0.7 µm intervals were acquired in Z using MetaXpress software (version revision 31201). 3D-reconstruction of Z-stack images and movies were done using the maximum intensity projection feature of ImageJ distribution Fiji (ImageJ 1.52). 16 fields of view were acquired for each timepoint. Time-lapses were obtained under the same imaging conditions, with a 2-second interval. Aggregate cross-sectional areas were measured in MATLAB. Briefly, intensity-based thresholding was used to define a binary mask that distinguished aggregates from their surroundings. The area, and the average signal intensity (proportional to the average cell density) was obtained for each segmented aggregate. The mixing velocity within aggregates of 12B01 was analyzed using the Matlab (2015b) plugin PIVlab (version 1.4) (2–4). The average velocity was determined over 5 frames (10 sec), using the Fast-Fourier-Transform algorithm with 3 passes (window sizes 32, 16, and 8 pixels).

2. Mathematical modeling of bacterial aggregates

The mathematical model describes individual cell activity within conceptual spherical aggregates in presence of radial chemical gradients. We develop an agent-based model to quantify single cell interactions with polymeric substrates including enzyme secretion, uptake of breakdown products, growth and division. The polymeric substrates diffuse into the aggregate from the aggregate periphery. The model assumes well-mixed conditions with no-accumulation of chemicals in the bulk environment (mimicking an open system) and thus the concentration of polymer and oligomer at aggregate periphery is modeled as a constant concentration. Oligomer concentration is assumed to be zero at the periphery, so polymer degradation within the aggregate provides the major oligomer for bacterial activity. Individual cells are uniform randomly distributed in the spherical domain but consume substrate and grow in response to local chemical conditions of oligomer concentration. Note that no motility is considered for the individual cells. Detailed description of the model is provided in the supplementary.

The following represents derivation of mathematical formulations and procedures to model microbial growth, enzymatic activity and diffusion processes.

2.1 Growth and division

In this model, the growth kinetics of individual cells, ν_s are dictated by Monod-type kinetics given as

$$\nu_i^s = B_i \frac{V_{\max}[\mathrm{C}]}{K_s + [\mathrm{C}]} \qquad \text{(S1)}$$

where B is the cell dry mass of an individual cell, *i* and $V_{\max}$ is the maximum substrate uptake and defined as: $V_{\max} = \mu_{\max} / Y_{\max}$ (maximum specific growth rate / substrate conversion yield to biomass). *Ks* is the half saturation constant for oligomer s. C_s is oligomer concentration and is assumed as the primary limiting substrate for the bacterial growth. We kept all other nutrients (e.g., oxygen, phosphate and nitrate) available at sufficient levels for microbial activity. Note that bacterial aggregates are often known to create anoxic microsites due to aerobic activity at their shells(5,6), however in our simulations the polymer concentration is kept low enough to avoid full oxygen depletion.

The actual biomass accumulation ($\frac{dB_i}{dt} = v_i^s Y_{\max}$) and maintenance of an individual cell ($\frac{dB_i}{dt} = B_i m_i Y_{\max}$) are linearly correlated with cell dry mass and therefore the new growth rate of individual cells (μ_{net}) is given as:

$$\mu_{net} = \left(\frac{v_i^{act}}{B_i} - m_i \right) Y_{\max} \qquad \text{(S2)}$$

In the individual based model, each cell may double to two daughter cells when a threshold amount of substrate has been taken up (7,8). The minimum volume of the individual cell at the threshold for the division ($V_{d,min}$) is given from the descriptive Donachie model (7,8)

$$V_{d,min} = 2\overline{V_u} / 1.433 \qquad \text{(S3)}$$

where $\overline{V_u}$ is median volume of the individual cell. The cell will divide into two identical (half-volume) cells if reaches $V_{d,min}$. In the simulation, the cylindrical cells are assumed. Under limited nutrient conditions, the actual growth rate of an individual cell is restricted by actual available amount of substrate within the domain (see (9,10)). The biological parameters of the growth kinetics are provided in Table S1.

2.2 Enzymatic activity

Extracellular enzymes at individual cell level is modeled by assuming that the enzyme is bound to external membranes of the cells. Bacterial cells depolymerize the local alginate polymers and releases low molecular weight substrates. Enzyme level ($S_{E,i}$) of a bacterial cell, *i* are assumed to be linearly correlated with cell biomass, B_i:

$$S_{E,i} = K_{enz.}B_i \qquad \text{(S4)}$$

$K_{enz.}$ is the fraction of biomass that is membrane bound enzyme and hydrolyzes polymeric substrates (enzyme production rate). We opted to use simplified version of individual based model (11)that assumes no-diffusion of enzymes that allows us to focus on the counter-gradients of oligomers and polysaccharide while uniform distribution of bacteria and their associated enzymes are guaranteed.

2.3 Oligomer and polymer diffusions along aggregate radius

The model explicitly simulates the diffusion oligomers and polymers within spherical aggregates. For oligomers, an absorbing condition (zero concentration) is assumed at external boundaries of the aggregates that simulates loss of oligomers to bulk environment. Diffusion of oligomers is modelled based on Fick's law of diffusion. We modeled a spherical volume of a single aggregate and diffusion of oligomers is assumed to be only in radial direction. Reaction-diffusion equation is then numerically solved by finite-difference method:

$$\frac{d[\mathrm{C}]}{dt} = K_P S_E(r).P(r) - B(r).V_{\max}.\frac{[\mathrm{C}](r)}{[\mathrm{C}](r)+\mathrm{K}_s} - D_{[\mathrm{C}]}^{eff.}(\varphi)\frac{1}{r^2}\frac{\partial}{\partial r}\left(r^2\frac{\partial[\mathrm{C}]}{\partial r}\right) \qquad \text{(S5)}$$

where [C] is the concentration of oligomers at radius, r. $K_{p.}$ is polymer lability that defines how many grams of oligomers are released per gram of enzyme acting on the polymer surface per unit of time. S_E is the enzyme amount and it is calculated from summing up the total enzymes of individual cells ($S_{E,i}$), presence in the corresponding radius ($S_E(r) = \sum_{i=1:n} S_{E,i}$, n is the number of individuals in the mesh grid at radius, r). B and P are the total biomass and polymer at radius, r, respectively. $D_{[\mathrm{C}]}^{eff.}$ is the effective diffusion coefficient of oligomers. To take into account the effects of cell density on the diffusion coefficient, we treated the bacterial aggregate as a porous environment in which cell density modifies aggregate porosity, ϕ. Porosity of the aggregate is calculated based on number of cells, volume of individual cells and size of the aggregate. We applied Millington equation to provide the relationship between porosity and effective diffusion coefficients (12):

$$D_{[\mathrm{C}]}^{eff.} \Big/ D_{[\mathrm{C}]} = \phi^{\frac{4}{3}} \qquad \text{(S6)}$$

$D_{[C]}$ is the diffusion coefficient of oligomers in bulk liquid.

Similar to oligomers, polymer diffusion is assumed to be only along the radial direction, as given:

$$\frac{d[\mathrm{P}]}{dt} = -K_P S_E(r).P(r) - D_{[\mathrm{P}]}^{eff.}(\varphi)\frac{1}{r^2}\frac{\partial}{\partial r}\left(r^2\frac{\partial[\mathrm{P}]}{\partial r}\right) \qquad \text{(S7)}$$

The physiological and chemical parameters used in the simulations are represented in Table S1.

2.4 Modeling biological strategies (mixing and aggregates with channels)

The main aggregate model is modified to implement biological responses on aggregate structure. In mixing strategy, the effective diffusion coefficients for both polymer and oligomers transport equations (Eq. S5 and S7) are enhanced to account for the effect of mixing on the transport.

To model aggregates with channels, we assumed that the channels are in equilibrium with the bulk environment and therefore the ultimate effect of the channeling is to subdivide the original aggregate structure into smaller individual sub-aggregates. We thus model the individual sub-aggregates as a separate unit and no interactions with other sub-aggregates are considered.

Table S1. Physiological and chemical parameters for microbial growth, metabolism and nutrient concentrations in the individual-based model.

Parameters	*Values (Units)*
μ_{max} : *maximum growth rate (hr^{-1})*	0.1*
K_s : *half saturation (mg/L)*	0.1*
Y_{max} : *growth yield (gr dry mass/gr substrate)*	0.5*
cell maintenance	0.1 μ_{max} ſ
cell size (μm)	1 ſ
ρ: cell density (mg L^{-1})	2.9×10^{5} ſ
V_{1D} *: cell velocity at bulk solution ($\mu m/s$)*	0*
V_u *: median cell volume (fl)*	0.4 ſ
K_p *polymer lability (hr^{-1})*	100 ꞁꞁ
$D_{[C]}$ *diffusion coefficient of oligomers in bulk liquid (m^{2}hr^{-1})*	2.4×10^{-6}
$D_{[p]}$· *Polymer diffusion coefficient in bulk liquid (m^{2}hr^{-1})*	1.7×10^{-5}

ſ (7)
| (13)
Τ (14)
ꞁꞁ*Enzyme Database – BRENDA: mean observed value for alginate*
**Model assumption*

Supplemental References

1. Hehemann J-H, Arevalo P, Datta MS, Yu X, Corzett CH, Henschel A, et al. Adaptive radiation by waves of gene transfer leads to fine-scale resource partitioning in marine microbes. Nat Commun [Internet]. The Author(s); 2016 Sep 22;7:12860. Available from: http://dx.doi.org/10.1038/ncomms12860
2. Thielicke W. The Flapping Flight of Birds - Analysis and Application. Rijksuniversiteit Groningen; 2014.
3. Thielicke, W. & Stamhuis EJ. PIVlab - Time-Resolved Digital Particle Image Velocimetry Tool for MATLABitle. 2014.
4. Thielicke, W. & Stamhuis EJ. PIVlab _ Towards User-friendly, Affordable and Accurate Digital Particle Image Velocimetry in MATLAB. J Open Res Softw. 2014;2(1):e30.
5. Ebrahimi A, Or D. Dynamics of soil biogeochemical gas emissions shaped by remolded aggregate sizes and carbon configurations under hydration cycles. Glob Chang Biol [Internet]. 2018 Jan 1;24(1):e378–92. Available from: http://dx.doi.org/10.1111/gcb.13938
6. Gärdes A, Iversen MH, Grossart HP, Passow U, Ullrich MS. Diatom-associated bacteria are required for aggregation of Thalassiosira weissflogii. ISME J. 2011;5(3):436–45.
7. Kreft JU, Booth G, Wimpenny JWT. BacSim, a simulator for individual-based modelling of bacterial colony growth. Microbiology. 1998;144(12):3275–87.
8. Ebrahimi AN, Or D. Microbial dispersal in unsaturated porous media: Characteristics of motile bacterial cell motions in unsaturated angular pore networks. Water Resour Res. 2014;50(9):7406–29.
9. Ebrahimi A, Or D. Hydration and diffusion processes shape microbial community organization and function in model soil aggregates. Water Resour Res [Internet]. 2015 Nov [cited 2015 Dec 2];51:9804–9827. Available from: http://doi.wiley.com/10.1002/2015WR017565
10. Ebrahimi A, Or D. Microbial community dynamics in soil aggregates shape biogeochemical gas fluxes from soil profiles – upscaling an aggregate biophysical model. Glob Chang Biol. 2016;22(9):3141–56.
11. Ebrahimi A, Schwartzman J, Cordero OX. Cooperation and spatial self-organization determine ecosystem function for polysaccharide-degrading bacteria. bioRxiv. 2019;
12. Millington RJ. Gas diffusion in porous media. Science (80-). 1959;
13. Kiørboe T, Grossart HP, Ploug H, Tang K. Mechanisms and rates of bacterial colonization of sinking aggregates. Appl Environ Microbiol. 2002;68(8):3996–4006.
14. Ahmed T, Stocker R. Experimental verification of the behavioral foundation of bacterial transport parameters using microfluidics. Biophys J. 2008;95(9):4481–93.
